# Sine Rotation Vector Method for Attitude Estimation of an Underwater Robot

**DOI:** 10.3390/s16081213

**Published:** 2016-08-02

**Authors:** Nak Yong Ko, Seokki Jeong, Youngchul Bae

**Affiliations:** 1 School of Electronic and Information Engineering, Chosun University, 375 Seosuk-dong Dong-gu, Gwangju 501-759, Korea; 2 Department of Control and Instrumentation Engineering, Graduate school, Chosun University, 375 Seosuk-dong Dong-gu, Gwangju 501-759, Korea; seokki@chosun.kr; 3 Division of Electrical, Electronic Communication and Computer Engineering, Chonnam National University, 50 Daehak-ro, Yeosu, Jeonnam 550-749, Korea; ycbae@chonnam.ac.kr

**Keywords:** underwater vehicle, underwater robot, attitude, sine rotation vector, Euler angles, attitude heading and reference system, Doppler velocity log

## Abstract

This paper describes a method for estimating the attitude of an underwater robot. The method employs a new concept of sine rotation vector and uses both an attitude heading and reference system (AHRS) and a Doppler velocity log (DVL) for the purpose of measurement. First, the acceleration and magnetic-field measurements are transformed into sine rotation vectors and combined. The combined sine rotation vector is then transformed into the differences between the Euler angles of the measured attitude and the predicted attitude; the differences are used to correct the predicted attitude. The method was evaluated according to field-test data and simulation data and compared to existing methods that calculate angular differences directly without a preceding sine rotation vector transformation. The comparison verifies that the proposed method improves the attitude estimation performance.

## 1. Introduction

Determining the attitude of underwater robots is one of the major research areas in the robotics field, because an underwater robot requires attitude information to estimate its location and control its motion [1]. The rotation and attitude with respect to a reference can be described in terms of Euler angles, quaternions, rotation matrices, rotation vectors and angle-axis representations. This paper proposes an attitude estimation approach that uses a new method called the sine rotation vector to represent rotations. If exteroceptive measurements of range to beacons and communication between the robot and beacons are available [2,3], it is possible to estimate the location without the information on attitude. However, for long-range navigation, it is practically not feasible to implement the system for range measurements or a communication network. Therefore, most underwater navigation methods require attitude data.

A vast number of studies have focused on the attitude estimation and navigation of underwater robots. The major approaches employ the following methodologies: methods using range measurement from acoustic beacons, methods integrating velocity over time, methods using landmarks or features of the environment, methods using range and bearing measurements and cooperative navigation. With the development of sensor technologies and data-fusion theory, methods that fuse a variety of heterogeneous sensors and theories have recently been developed. The navigation methods for underwater robots comprise aspects that are similar to the methods used for ground-robot navigation, whereby the multiple measurements of different features are fused [4,5].

The Kalman filter approach is used in many methods designed for the attitude estimation and navigation of underwater robots. The nonlinear dynamics, forces, moments, position and velocity of the robot are fused for the estimation through a nonlinear observer and an extended Kalman filter (EKF) [6]. A method that uses a robot kinetic model has also been proposed [7]. This method, which can also be used to estimate sea currents, makes full use of the measurements from inertial navigation systems (INS) or attitude heading and reference systems (AHRS), ultrashort base line (USBL) systems and a Doppler velocity log (DVL) for the purpose of navigation. Another similar approach that uses robot dynamics for navigation, integrating low-cost INS and USBL systems, has also been reported [8]. Notably, an adaptive dynamic model and an EKF are fused for the autonomous navigation of underwater robots [9].

A navigation method that also estimates the sensor parameters has been proposed. The method uses an inertial measurement unit (IMU), a DVL, a magnetic compass and a depth sensor, all of which constitute a typical set of navigation sensors [10]. Similar to the method described in [7], a method that concurrently estimates the location of a robot and the sea level has also been proposed [11]. Besides the popular Kalman filter-based approaches [12], some methods adopt particle filters (PFs) for the estimation [13,14].

Interference of the magnetic field is one of the most critical issues when using a magnetometer for attitude estimation. Though many methods have investigated the estimation of the bias of the magnetic field measurement [15], it is still hard to estimate and compensate for the bias because of the following reasons: the bias and interference depend on the environmental aspects, which are varying both with time and place; and it is in all directions, that is the bias is in the directions of north, east and Earth-center.

The proposed method does not explicitly estimate and compensate for the bias and interference of the magnetic field measurement. Nevertheless, the sine rotation vector method reduces the effect of bias and interference. The method uses only the northward direction of the magnetic field, and ignores the east and Earth-center direction components. Therefore, it is not affected by the bias and interference in these two directions. Likewise, the method uses only the Earth-center direction of the acceleration, thus eliminating the interference affecting the acceleration measurement toward the north and east.

This paper proposes a new approach called the sine rotation vector method, whereby an EKF is applied to estimate the attitude of an underwater robot. In some of the previous methods, the measurement innovation or residual is calculated by simply subtracting predicted Euler angles from the measured Euler angles in the correction step; however, it should be noted that physical rotation is not linearly related to Euler angles.

Euler angles can be used for the representation of both the rotation and attitude. However, the difference of two attitudes represented in Euler angles does not necessarily represent the Euler angles needed for the rotation between the attitudes. It is generally understood that the relationship $R\left(\phi_{1},\theta_{1},\psi_{1}\right)R\left(\phi_{2},\theta_{2},\psi_{2}\right)=R\left(\phi_{1}+\phi_{2},\theta_{1}+\theta_{2},\psi_{1}+\psi_{2}\right)$ does not hold, where R(ϕ,θ,ψ) is the rotation matrix with the roll *φ*, pitch *θ* and yaw *ψ*. To avoid the problem, the sine rotation vector is used to represent the rotation instead of the direct difference of Euler angles.

In general, correspondence between the Euler angle difference and the physical rotation is not uniquely determined. The difference between the Euler angles that represent two different attitudes does not indicate the rotation between the two attitudes. Let $x_{1}$ and $x_{2}$ indicate two attitudes that are represented by Euler angles. The rotation from attitude $x_{1}$ to attitude $x_{2}$ is not necessarily the rotation that is indicated by the Euler angle difference calculated by subtracting $x_{1}$ from $x_{2}$. The difference between the Euler angles is therefore not a legitimate representation of the measurement innovation; that is, the difference between the Euler angles does not indicate the rotation needed to adjust the predicted attitude for the Kalman filter correction step. The difference between the Euler angles of the measured attitude and the predicted attitude does not represent the physical relationship between the measured attitude and the predicted attitude. To remove the ambiguity in the calculation of the measurement innovation, the proposed method computes the innovation by calculating the sine rotation vector from the predicted attitude to the measured attitude and then converting the sine rotation vector to Euler angles. This method produces a unique and physically-consistent measurement innovation that corresponds to the pair composed of the measured attitude and the predicted attitude.

Quaternions and SO(3) matrices have been used for attitude estimation [16,17,18]. The quaternion and SO(3) matrix uniquely determine the rotation required to rotate the predicted attitude to the measured attitude as the sine rotation vector does. The problem to be addressed in using the quaternion is keeping the magnitude of the quaternion to one [19,20]. In the stage of prediction and correction, the magnitude of the quaternion deviates from one. Therefore, it is required to use some subterfuge to normalize the quaternion. Likewise, the SO(3) matrix should have the property of orthogonality. Therefore, the methods using the SO(3) matrix inevitably use some method to approximate the predicted and corrected matrix to some orthogonal matrix [19,20]. The use of the sine rotation vector does not require approximation for the normalization of vectors or the orthogonalization of matrices.

The attitude estimation problem studied in this paper is formulated in Section 2. Section 3, Section 4 and Section 5 describe the attitude estimation procedure. Section 6 shows the results of experiments and simulations using the proposed method and the previously-reported methods and presents a comparison of these results. Section 7 concludes the paper and suggests further research regarding the proposed method.

## 2. Problem Formulation

### 2.1. Nomenclature

The notations that are used for the derivation of the method are listed below.


x(t)
attitude of a robot at time *t*; $x\left(t\right)={\left(\phi (t),\theta (t),\psi (t)\right)}^{T}$, where ϕ(t), θ(t) and ψ(t) indicate roll, pitch and yaw
$\hat{x}\left(t\right)$
attitude estimated at time *t* through prediction and a correction procedure; $\hat{x}\left(t\right)={\left(\hat{\phi }\left(t\right),\hat{\theta }\left(t\right),\hat{\psi }\left(t\right)\right)}^{T}$
$\hat{P}\left(t\right)$
error covariance of the estimated attitude $\hat{x}\left(t\right)$
$x^{-}\left(t\right)$
attitude predicted at time *t*, before it is corrected by the measurements; $x^{-}\left(t\right)={\left(\phi^{-}\left(t\right),\theta^{-}\left(t\right),\psi^{-}\left(t\right)\right)}^{T}$
$P^{-}\left(t\right)$
error covariance of the predicted attitude $x^{-}\left(t\right)$
a(t)
acceleration measured in the instrument coordinate frame; $a\left(t\right)={\left(a_{x}\left(t\right),a_{y}\left(t\right),a_{z}\left(t\right)\right)}^{T}$

$a_{unit}\left(t\right)$
normalized acceleration measurement; $a_{unit}\left(t\right)={\left(a_{x}\left(t\right),a_{y}\left(t\right),a_{z}\left(t\right)\right)}_{unit}^{T}=\frac{a(t)}{∥a(t)∥}$

m(t)
magnetic field measured in the instrument coordinate frame; $m\left(t\right)={\left(m_{x}\left(t\right),m_{y}\left(t\right),m_{z}\left(t\right)\right)}^{T}$

$m_{unit}\left(t\right)$
normalized magnetic field measurement; $m_{unit}\left(t\right)={\left(m_{x}\left(t\right),m_{y}\left(t\right),m_{z}\left(t\right)\right)}_{unit}^{T}=\frac{m(t)}{∥m(t)∥}$

z(t)
measurements of roll, pitch and yaw calculated from the a(t) and m(t) at time *t*; $z\left(t\right)={\left(\phi \left(t\right),\theta \left(t\right),\psi \left(t\right)\right)}^{T}$
v(t)
linear velocity of the robot in the robot coordinate frame; $v\left(t\right)={\left(u(t),v(t),w(t)\right)}^{T}$
ω(t)
rotational velocity of the robot in the robot coordinate frame; $\omega \left(t\right)={\left(p\left(t\right),q\left(t\right),r\left(t\right)\right)}^{T}$
g(·)
motion model of a robot that relates the robot attitude x(t) and the linear velocity v(t) to the rotational velocity $\dot{x}\left(t\right)$ of the robot; $\dot{x}\left(t\right)=g\left(x(t),v(t)\right)$
h(·)
the measurement model that relates state x(t) to the measurement z(t); z(t)=h(x(t))
$t_{k}$
the *k*-th discretized sampling time instant
$\Delta t_{k}$
time period between $t=t_{k-1}$ and $t=t_{k}$; $\Delta t_{k}=t_{k}-t_{k-1}$

In many studies, an underwater robot is often referred to as an underwater vehicle. Thus, in the following sections, the terms vehicle and robot will be used interchangeably.

### 2.2. Problem Formulation

The following is a description of the problem to be solved.

**Attitude** **estimation problem:** Find the attitude $\hat{x}\left(t_{k}\right)$ of an underwater robot at time $t=t_{k}$, given the provided measurements $a(t_{k})$, $m(t_{k})$. The attitude $\hat{x}\left(t_{k-1}\right)$ estimated at time $t=t_{k-1}$, the linear velocity $v(t_{k})$ and the angular velocity $\omega (t_{k})$ are given.

The estimation procedure repeats at every time $t_{k}$ when new measurements are available, and it runs recursively based on the results provided at the previous estimation sequence. The estimated attitude $\hat{x}\left(t_{k}\right)$ will be used for the estimation of $\hat{x}\left(t_{k+1}\right)$ at the next iteration of the procedure. The estimation procedure consists of two sub-procedures: prediction and correction. The prediction and correction procedures will be derived in the following sections.

The rotational velocity of the vehicle $\omega (t_{k})$ detected with a gyroscope in the AHRS is used for the prediction. The velocity $\omega (t_{k})$ is measured in the instrument coordinate frame. It is assumed that the instrument coordinate frame is adjusted to coincide with the vehicle coordinate frame. The acceleration $a(t_{k})$ and magnetic field $m(t_{k})$ measurements are used for the correction; furthermore, these are measured by an AHRS in the instrument coordinate frame that is also assumed to be coincident with the vehicle coordinate frame.

The linear velocity of the vehicle $v(t_{k})$ is detected with the DVL. The velocity $v(t_{k})$ is measured in the instrument coordinate frame that also coincides with the vehicle coordinate frame. Using the estimated attitude, the linear velocity $v(t_{k})$ is converted to the velocity in the Earth-fixed coordinate frame; this converted velocity is then integrated to find the location of the robot in the Earth-fixed coordinate frame. The linear velocity $v(t_{k})$ can also be used to extract the pure gravitation field by removing the vehicle acceleration from the acceleration measurement $a(t_{k})$.

The proposed method utilizes the short-term superiority of the angular rate measurement and the long-term stability of the attitude algebraically calculated from the acceleration and magnetic field measurement. Table 1 depicts the complementary aspects of the these measurements. The short-term superiority of the gyroscope measurement is utilized in the prediction stage where the attitude is predicted using the angular rate measurement. Though the angular rate provides the short-term change of the attitude with high accuracy, the accuracy of the attitude calculated from the numerical integration of the attitude change deteriorates with time. To compensate for the deterioration, the long-term stability of the attitude that is algebraically calculated from the acceleration and the magnetic field is used in the measurement update or correction stage. The DVL is used to calculate the location using the estimated attitude. Furthermore, it can be used to reduce the system dynamics effect that perturbs the gravitation field in calculating the roll and pitch of the robot.

## 3. Predictions of Attitude and Covariance

The prediction step that is otherwise called the “time update step” is used to predict the state and error covariances for time $t_{k}$; to complete the step, the measured angular velocity $\omega (t_{k-1})$ at time $t=t_{k-1}$, the state $\hat{x}\left(t_{k-1}\right)$ and the error covariance $\hat{P}\left(t_{k-1}\right)$ that was estimated previously at time $t_{k-1}$ are used. The prediction is based on the function g(·) that is used to determine the time derivative of the attitude, as follows:

(1) $$\dot{x}\left(t_{k}\right)=g\left(x\left(t_{k}\right),v\left(t_{k}\right)\right)$$

(2) $$\left(\begin{array}{c} \dot{\phi }\left(t_{k}\right)\\ \dot{\theta }\left(t_{k}\right)\\ \dot{\psi }\left(t_{k}\right) \end{array}\right)=\underset{g(x\left(t_{k}\right),v\left(t_{k}\right))}{\underset{︸}{\left(\begin{array}{c} p\left(t_{k}\right)+q\left(t_{k}\right)S\left(\hat{\phi }\left(t_{k}\right)\right)T\left(\hat{\theta }\left(t_{k}\right)\right)+r\left(t_{k}\right)C\left(\hat{\phi }\left(t_{k}\right)\right)T\left(\hat{\theta }\left(t_{k}\right)\right)\\ q\left(t_{k}\right)C\left(\hat{\phi }\left(t_{k}\right)\right)-r\left(t_{k}\right)S\left(\hat{\phi }\left(t_{k}\right)\right)\\ q\left(t_{k}\right)S\left(\hat{\phi }\left(t_{k}\right)\right)\sec\left(\hat{\theta }\left(t_{k}\right)\right)+r\left(t_{k}\right)C\left(\hat{\phi }\left(t_{k}\right)\right)\sec\left(\hat{\theta }\left(t_{k}\right)\right) \end{array}\right)}}$$

Using Equations (1) and (2), the attitude is predicted as $x^{-}\left(t_{k}\right)$ according to Equations (3) and (4), as follows:

(3) $$x^{-}\left(t_{k}\right)=\hat{x}\left(t_{k-1}\right)+\dot{x}\left(t_{k-1}\right)\Delta t_{k}$$

(4) $$\left(\begin{array}{c} \phi^{-}\left(t_{k}\right)\\ \theta^{-}\left(t_{k}\right)\\ \psi^{-}\left(t_{k}\right) \end{array}\right)=\left(\begin{array}{c} \hat{\phi }\left(t_{k-1}\right)+\dot{\phi }\left(t_{k-1}\right)\Delta t_{k}\\ \hat{\theta }\left(t_{k-1}\right)+\dot{\theta }\left(t_{k-1}\right)\Delta t_{k}\\ \hat{\psi }\left(t_{k-1}\right)+\dot{\psi }\left(t_{k-1}\right)\Delta t_{k} \end{array}\right)$$

where $\Delta t_{k}=t_{k}-t_{k-1}$. In these equations, S(·), C(·) and T(·) represent sin(·), cos(·) and tan(·), respectively.

The Jacobian matrix J of the function g(·) is used to find the linearized state transition matrix A according to Equation (5), as follows:

(5) $$A=I_{3\times 3}+\Delta t\;J$$

The Jacobian matrix J of the function g(·) is given according to Equation (6), as follows:

(6) $$J=\frac{\partial g}{\partial x}\left(\hat{x}\left(t_{k-1}\right),v\left(t_{k-1}\right)\right)=\left(\begin{array}{ccc} \left(qC\left(\hat{\phi }\right)-rS\left(\hat{\phi }\right)\right)T\left(\hat{\theta }\right) & \left(qS\left(\hat{\phi }\right)+rC\left(\hat{\phi }\right)\right)\sec^{2}\left(\hat{\theta }\right) & 0\\ -qS\left(\hat{\phi }\right)-rC\left(\hat{\phi }\right) & 0 & 0\\ \left(qC\left(\hat{\phi }\right)-rS\left(\hat{\phi }\right)\right)\sec\left(\hat{\theta }\right) & \left(qS\left(\hat{\phi }\right)+rC\left(\hat{\phi }\right)\right)\sec\left(\hat{\theta }\right)T\left(\hat{\theta }\right) & 0 \end{array}\right)$$

where the time variable $\left(t_{k-1}\right)$ is deleted from *p*, *q*, *r*, $\hat{\phi }$ and $\hat{\theta }$ for notational simplicity.

The error covariance $P^{-}\left(t_{k}\right)$ of the predicted attitude $x^{-}\left(t_{k}\right)$ is derived as Equation (7) through the use of the linearized state transition matrix A, as follows:

(7) $$P^{-}\left(t_{k}\right)=A\hat{P}\left(t_{k-1}\right)A^{T}+R$$

where R is the error covariance of the vehicle motion. The two Equations (4) and (7) complete the prediction step.

## 4. Corrections of Predicted Attitude and Covariance

The correction step that is otherwise called the “measurement update step” adjusts the predicted attitude $x^{-}\left(t_{k}\right)$ and error covariance $P^{-}\left(t_{k}\right)$ by using the measurements at time $t_{k}$, $z(t_{k})$. For the corrections, the measurement model matrix H that is the linearized measurement model of h(·) is required. Because the measurement $z(t_{k})$ consists of the roll, pitch and yaw of the Euler angles that match those in state $x(t_{k})$, the measurement model matrix H is identity, that is H=I.

Using the measurement model H, the Kalman gain for the adjustment of $x^{-}\left(t_{k}\right)$ and $P^{-}\left(t_{k}\right)$ is given according to Equation (8), as follows:

(8) $$K\left(t_{k}\right)=P^{-}\left(t_{k}\right)H^{T}{\left(HP^{-}\left(t_{k}\right)H^{T}+Q\right)}^{-1}$$

where Q is the error covariance of the measurement.

The predicted attitude $x^{-}\left(t_{k}\right)$ is corrected to $\hat{x}\left(t_{k}\right)$ by Equation (9), as follows:

(9) $$\hat{x}\left(t_{k}\right)=x^{-}\left(t_{k}\right)+K\left(t_{k}\right)\left(z\left(t_{k}\right)-h\left(x^{-}\left(t_{k}\right)\right)\right)$$

The predicted covariance $P^{-}\left(t_{k}\right)$ is adjusted to $\hat{P}\left(t_{k}\right)$ by Equation (10), as follows:

(10) $$\hat{P}\left(t_{k}\right)=P^{-}\left(t_{k}\right)-K\left(t_{k}\right)HP^{-}\left(t_{k}\right)$$

The innovation $\nu \left(t_{k}\right)=z\left(t_{k}\right)-h\left(x^{-}\left(t_{k}\right)\right)$ in Equation (9) will be derived in a manner that is different from the usual methods. The proposed method that uses the sine rotation vector will be described in Section 5.

## 5. Innovation by Sine Rotation Vector

### 5.1. Calculation of Innovation by Sine Rotation Vector

The innovation $\nu (t_{k})$ is given by Equation (11), as follows:

(11) $$\nu \left(t_{k}\right)=z\left(t_{k}\right)-h\left(x^{-}\left(t_{k}\right)\right)$$

The innovation $\nu (t_{k})$ is the difference between the measured attitude $z(t_{k})$ and the presumed measurement $h(x^{-}\left(t_{k}\right))$ that is calculated under the assumption that the robot is in its predicted attitude $x^{-}\left(t_{k}\right)$. Many existing methods calculate the measurement $z(t_{k})$ from the acceleration $a(t_{k})$ and magnetic field $m(t_{k})$, whereby the predicted attitude $x^{-}\left(t_{k}\right)$ is used as the presumed measurement $h(x^{-}\left(t_{k}\right))$.

This paper proposes a new approach for finding the innovation, which is the difference between the measured attitude $z(t_{k})$ and the presumed attitude $h(x^{-}\left(t_{k}\right))$. The proposed method finds the sine rotation vector between the presumed attitude and the measured attitude. Because vehicle acceleration is negligible compared to gravity, the measured acceleration $a(t_{k})$ is directed toward the −z direction of the Earth-fixed coordinate frame; that is, the acceleration measurement $a(t_{k})$ is the Earth-fixed −z direction vector that is represented in the instrument coordinate frame. Let the presumed −z direction represented in the vehicle coordinate frame be denoted by $-z_{pres}\left(t_{k}\right)$. The presumed −z direction vector $-z_{pres}\left(t_{k}\right)$ is calculated according to Equation (12), as follows:

(12) $$-z_{pres}\left(t_{k}\right)={}^{v}R_{w}\left(t_{k}\right){\left(0,0,-1\right)}^{T}$$

where ${}^{v}R_{w}\left(t_{k}\right)$ is the rotation matrix from the Earth-fixed coordinate frame to the vehicle coordinate frame at time $t=t_{k}$. ${}^{v}R_{w}\left(t_{k}\right)$ is calculated using the predicted attitude $x^{-}\left(t_{k}\right)$; then, the cross product of $-z_{pres}\left(t_{k}\right)$ and $a_{unit}\left(t_{k}\right)$ meets the sine rotation vector $r_{-z}\left(t_{k}\right)$. The sine rotation vector $r_{-z}\left(t_{k}\right)$ represents the rotation from the presumed −z direction to the measured −z direction, as follows:

(13) $$r_{-z}\left(t_{k}\right)=-z_{pres}\left(t_{k}\right)\times a_{unit}\left(t_{k}\right)$$

**Figure 1:**
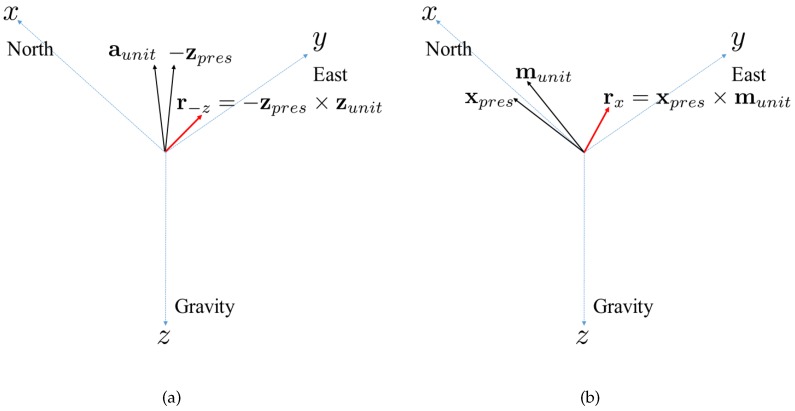
Sine rotation vectors. (**a**) Sine rotation vector rotating *z* directional vectors; (**b**) Sine rotation vector rotating *x* directional vectors.

The direction of the sine rotation vector $r_{-z}\left(t_{k}\right)$ represents the axis of rotation from the presumed −z direction to the measured −z direction. The length of the sine rotation vector $r_{-z}\left(t_{k}\right)$ is the sine of the rotation angle from the presumed −z direction to the measured −z direction. Figure 1a represents graphically how the $r_{-z}\left(t_{k}\right)$ is calculated and represents the rotation.

Likewise, the measured magnetic field $m(t_{k})$ directs the Earth-fixed *x* direction that is represented in the instrument coordinate frame. The bias of the measured magnetic field $m(t_{k})$ from the Earth-fixed *x* direction should be adjusted by using the methods for bias estimation [15,21]. Let the presumed *x* direction that is represented in the vehicle coordinate frame be denoted by $x_{pres}\left(t_{k}\right)$. Then, Equation (14) provides the presumed *x* direction vector $x_{pres}\left(t_{k}\right)$ that is represented in the vehicle coordinate frame, as follows:

(14) $$x_{pres}\left(t_{k}\right)={}^{v}R_{w}\left(t_{k}\right){\left(1,0,0\right)}^{T}$$

The cross product of $x_{pres}\left(t_{k}\right)$ and $m_{unit}\left(t_{k}\right)$ then becomes the sine rotation vector $r_{x}\left(t_{k}\right)$. The sine rotation vector $r_{x}\left(t_{k}\right)$ represents the rotation from the presumed *x* direction to the measured *x* direction, as follows:

(15) $$r_{x}\left(t_{k}\right)=x_{pres}\left(t_{k}\right)\times m_{unit}\left(t_{k}\right)$$

Figure 1b represents graphically how the $r_{x}\left(t_{k}\right)$ is calculated and represents the rotation.

Both of the sine rotation vectors $r_{-z}\left(t_{k}\right)$ and $r_{x}\left(t_{k}\right)$ bear the rotation information from the presumed attitude to the measured attitude. These two vectors are the rotations determined by two different sensors (the accelerometer and the magnetometer) and are combined using Equation (16), as follows:

(16) $$r\left(t_{k}\right)=\gamma_{-z}r_{-z}\left(t_{k}\right)+\gamma_{x}r_{x}\left(t_{k}\right)$$

$\gamma_{-z}$ and $\gamma_{x}$ in Equation (16) are mixing parameters with a sum value of one, as follows:

(17) $$\gamma_{-z}+\gamma_{x}=1$$

The values of $\gamma_{-z}$ and $\gamma_{x}$ need to be set based on the general rule: the larger value is assigned to the *γ* that corresponds to the more reliable measurement between the acceleration and magnetic field. If the acceleration measurement is more reliable than the magnetic field measurement, then $\gamma_{-z}$ will have a larger value, and vice versa. In the experiment, the values are determined through trial and error, as well as based on the general rule.

From the sine rotation vector, the rotation axis b and rotation angle *β* are found by using Equations (18) and (19), as follows:

(18) $$b={\left(b_{1},b_{2},b_{3}\right)}^{T}=\frac{r(t_{k})}{∥r(t_{k})∥}$$

(19) $$\beta =\sin^{-1}(∥r\left(t_{k}\right)∥)$$

The rotation matrix R(β,b) that is represented in terms of the rotation angle and rotation axis is described in Equation (20), as follows:

(20) $$R\left(\beta ,b\right)=\left(\begin{array}{ccc} b_{1}^{2}V\left(\beta \right)+C\left(\beta \right)\; & \;b_{1}b_{2}V\left(\beta \right)-b_{3}S\left(\beta \right)\; & \;b_{1}b_{3}V\left(\beta \right)+b_{2}S\left(\beta \right)\\ b_{1}b_{2}V\left(\beta \right)+b_{3}S\left(\beta \right) & b_{2}^{2}V\left(\beta \right)+C\left(\beta \right) & b_{2}b_{3}V\left(\beta \right)-b_{1}S\left(\beta \right)\\ b_{1}b_{3}V\left(\beta \right)-b_{2}S\left(\beta \right)\; & \;b_{2}b_{3}V\left(\beta \right)+b_{1}S\left(\beta \right) & b_{3}^{2}V\left(\beta \right)+C\left(\beta \right) \end{array}\right)$$

where V(β)=1−C(β) is applied. The rotation matrix can also be represented in terms of the Euler angles (ϕ,θ,ψ) as the matrix R(ϕ,θ,ψ), which is shown in Equation (21).

(21) $$\begin{array}{cc} R(\phi ,\theta ,\psi )= & \left(\begin{array}{cc} C(\theta )C(\psi )\; & \;S(\phi )S(\theta )C(\psi )-C(\phi )S(\psi )\\ C(\theta )S(\psi )\; & \;S(\phi )S(\theta )S(\psi )+C(\phi )C(\psi )\\ -S(\theta ) & S(\phi )C(\theta ) \end{array}\right.\\ & \left.\begin{array}{c} C(\phi )S(\theta )C(\psi )+S(\phi )S(\psi )\\ C(\phi )S(\theta )S(\psi )-S(\phi )C(\psi )\\ C(\phi )C(\theta ) \end{array}\right) \end{array}$$

From the equivalence of the two rotation matrices R(β,b) and R(ϕ,θ,ψ), the Euler angles are found by using Equation (22), as follows.

(22) $$\begin{array}{cc} \left(\begin{array}{c} \phi \\ \theta \\ \psi \end{array}\right) & =\left(\begin{array}{c} \mathrm{atan}2(R_{32},R_{33})\\ \mathrm{atan}2(-R_{31},\sqrt{R_{11}^{2}+R_{22}^{2}})\\ \mathrm{atan}2(R_{21},R_{11}) \end{array}\right)\\ & =\left(\begin{array}{c} \mathrm{atan}2\left(b_{2}b_{3}V\left(\beta \right)+b_{1}S\left(\beta \right),b_{3}^{2}V\left(\beta \right)+C\left(\beta \right)\right)\\ \mathrm{atan}2(-b_{1}b_{3}V\left(\beta \right)+b_{2}S\left(\beta \right),\sqrt{{\left(b_{1}^{2}V\left(\beta \right)+C\left(\beta \right)\right)}^{2}+{\left(b_{2}^{2}V\left(\beta \right)+C\left(\beta \right)\right)}^{2}}\\ \mathrm{atan}2(b_{1}b_{2}V\left(\beta \right)+b_{3}S\left(\beta \right),b_{1}^{2}V\left(\beta \right)+C\left(\beta \right)) \end{array}\right) \end{array}$$

The Euler angles in Equation (22) represent the rotation from the presumed attitude to the measured attitude; consequently, it is concluded that the innovation $\nu \left(t_{k}\right)=z\left(t_{k}\right)-h\left(x^{-}\left(t_{k}\right)\right)$ that was used for Equation (9) is found by using Equation (23), as follows:

(23) $$\begin{array}{cc} \nu (t_{k}) & =z\left(t_{k}\right)-h\left(x^{-}\left(t_{k}\right)\right)\\ & =\left(\begin{array}{c} \mathrm{atan}2\left(b_{2}b_{3}V\left(\beta \right)+b_{1}S\left(\beta \right),b_{3}^{2}V\left(\beta \right)+C\left(\beta \right)\right)\\ \mathrm{atan}2(-b_{1}b_{3}V\left(\beta \right)+b_{2}S\left(\beta \right),\sqrt{{\left(b_{1}^{2}V\left(\beta \right)+C\left(\beta \right)\right)}^{2}+{\left(b_{2}^{2}V\left(\beta \right)+C\left(\beta \right)\right)}^{2}}\\ \mathrm{atan}2(b_{1}b_{2}V\left(\beta \right)+b_{3}S\left(\beta \right),b_{1}^{2}V\left(\beta \right)+C\left(\beta \right)) \end{array}\right) \end{array}$$

A brief explanation regarding the calculation of the measurement $z={\left(\phi ,\theta ,\psi \right)}^{T}$ from the acceleration $a={\left(a_{x},a_{y},a_{z}\right)}^{T}$ and magnetic field $m={\left(m_{x},m_{y},m_{z}\right)}^{T}$ follows [22]. First, the roll *φ* and pitch *θ* are calculated using the acceleration $a={\left(a_{x},a_{y},a_{z}\right)}^{T}$ in Equations (24) and (25), as follows:

(24) $$\phi =\mathrm{atan}2(-a_{y},-a_{z})$$

(25) $$\theta =\mathrm{atan}2(a_{x},\sqrt{a_{y}^{2}+a_{z}^{2}})$$

*φ* and *θ* are used to obtain the transformation matrix ${}^{w}R_{v}$ through the use of Equation (26), as follows:

(26) $${}^{w}R_{v}\left(\phi ,\theta \right)=\left(\begin{array}{ccc} C(\theta )\; & \;S(\phi )S(\theta )\; & \;C(\phi )S(\theta )\\ 0 & -C(\theta ) & -S(\phi )\\ -S(\theta )\; & \;S(\phi )C(\theta )\; & \;C(\phi )C(\theta ) \end{array}\right)$$

Then, the yaw *ψ* is calculated using Equation (27), as follows:

(27) $$\begin{array}{c} \psi =\mathrm{atan}2({-}^{w}m_{y}{,}^{w}m_{z})\\ \mathrm{where},\;{\;}^{w}m={{(}^{w}m_{x}{,}^{w}m_{y}{,}^{w}m_{z})}^{T}{=}^{w}R_{v}\left(\phi ,\theta \right)m \end{array}$$

### 5.2. Sine Rotation Vector

The sine rotation vector is defined by the following Equation (28).

(28) r=(sinβ)b

where b is the unit vector in the direction of the rotation axis and *β* is the angle of rotation. The sine rotation vector is different from the rotation vector, which is defined by rb=βb [20,23]. The difference between the sine rotation vector and rotation vector is the magnitude of the vectors. The magnitude of the sine rotation vector is the sine of the rotation angle, while the magnitude of the rotation vector is the rotation angle itself.

The use of the sine rotation vector improves the attitude estimation in calculating the innovation vector in the EKF measurement update stage. First, the sine rotation vector uniquely determines the physical rotation regardless of the attitude to be rotated. In the case of the Euler angle difference between two attitudes represented in Euler angles, the rotation by the Euler angle difference does not necessarily correspond to the rotation between the attitudes. For example, the rotation of the attitude $\left(\phi_{1},\theta_{1},\psi_{1}\right)=\left(0,\frac{\pi }{2},\frac{\pi }{3}\right)$ by the rotation $\left(\phi_{2},\theta_{2},\psi_{2}\right)=\left(\frac{\pi }{6},\frac{\pi }{6},\frac{\pi }{6}\right)$ does not result in the attitude $\left(\phi_{1}+\phi_{2},\theta_{1}+\theta_{2},\psi_{1}+\psi_{2}\right)=\left(0+\frac{\pi }{6},\frac{\pi }{2}+\frac{\pi }{6},\frac{\pi }{3}+\frac{\pi }{6}\right)$. On the other hand, the rotation of the attitude $\left(\phi_{1},\theta_{1},\psi_{1}\right)=\left(0,0,0\right)$ by the rotation $\left(\phi_{2},\theta_{2},\psi_{2}\right)=\left(\frac{\pi }{6},\frac{\pi }{6},\frac{\pi }{6}\right)$ does result in the attitude $\left(\phi_{1}+\phi_{2},\theta_{1}+\theta_{2},\psi_{1}+\psi_{2}\right)=\left(0+\frac{\pi }{6},0+\frac{\pi }{6},0+\frac{\pi }{6}\right)$. This example illustrates that the same rotation results in different physical rotation depending on the attitude to which the rotation is applied. On the contrary, the sine rotation vectors $r_{x}$ and $r_{-z}$ uniquely rotate the predicted directions $x_{pres}$ and $-z_{pres}$ to the measured directions $m_{unit}$ and $a_{unit}$, regardless of the attitudes. In summary, the sine rotation vector uniquely determines the physical rotation regardless of the attitude to be rotated.

Second, the sine rotation vector relieves the effect of the bias and interference in measurement of the gravitational field and the magnetic field. The sine rotation vectors $r_{x}$ and $r_{-z}$ deal only with *x* and −z directional measurements, respectively. $r_{x}$ is free from the bias in the direction of *y* and *z*. Likewise, $r_{-z}$ is free from the bias in the direction of *x* and *y*. Therefore, use of the sine rotation vector mitigates the effect of bias in the field measurement, even though the method does not explicitly estimate and compensate the bias.

## 6. Experiments and Results

The method is tested in simulations and two experimental environments. The simulation uses two measurement datasets, which are subject to both random Gaussian noise and bias. The first experiment uses an underwater robot in a test pool. The second test uses a ground vehicle equipped with GPS, AHRS and a high-precision FOG (fiber optic gyroscope). The results were analyzed and compared to the results of other methods that calculate innovation $\nu (t_{k})$ by using the Euler angles of Equations (24), (25) and (27), without transforming to the sine rotation vector; these other methods include EKF, the unscented Kalman filter (UKF) and the complementary filter (CF).

### 6.1. Test through Simulation

The sine rotation vector method is tested using simulation data. For comparison, EKF, which calculates innovation by subtracting the measured Euler angles from the predicted Euler angles without transforming to the sine rotation vector, is also implemented. Two simulated test datasets, which differ in measurement error, are used. They assume the same robot motion, where the robot moves with the linear velocity and angular velocity given as Equations (29) and (30), over the traveled distance 600 m for the time period of 600 s.

(29) $$\left(\begin{array}{c} u(t)\\ v(t)\\ w(t) \end{array}\right)=\left(\begin{array}{c} 1m/s\\ 0m/s\\ 0m/s \end{array}\right)$$

(30) $$\left(\begin{array}{c} p(t)\\ q(t)\\ r(t) \end{array}\right)=\left(\begin{array}{c} 0.03\pi \sin(t/10)rad/s\\ 0.03\pi \cos(t/10)rad/s\\ 0.03\pi \sin(t/100)rad/s \end{array}\right)$$

As for the measurement of linear velocity by a DVL, the measurement is assumed to have Gaussian noise with zero mean and σ=0.2 m of standard deviation in all of the *x*, *y* and *z* directions. The measurement data for acceleration and magnetic field are generated by simulation, such that the attitude includes some bias, as well as random Gaussian noise. Table 2 describes the bias and random noise in attitude applied to produce the two simulated measurement datasets. Measurement data for Simulation 1 are affected more by bias than the data for Simulation 2, while they are corrupted by the same degree of random Gaussian noise.

**Figure 2:**
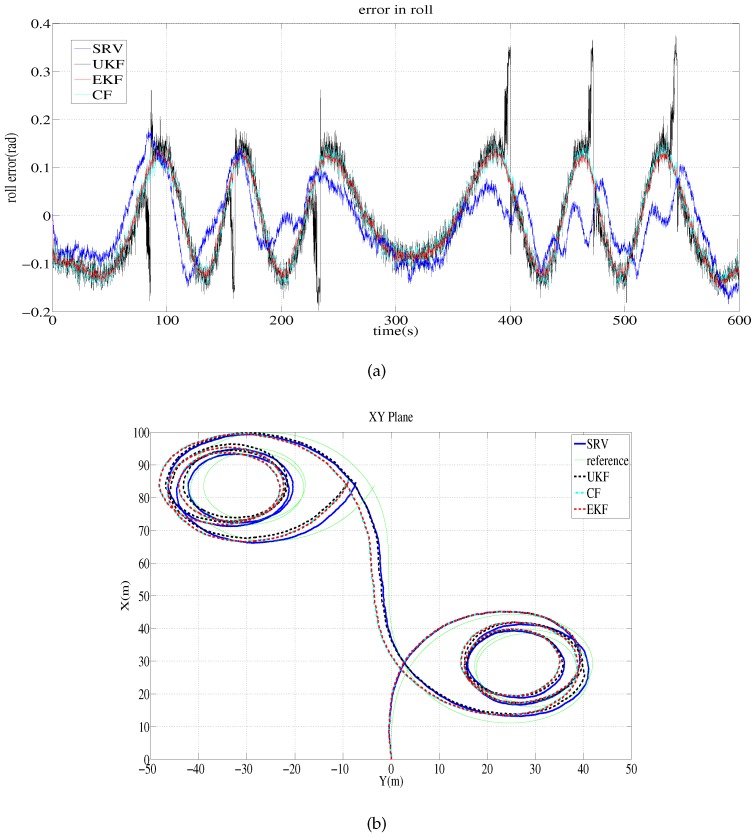
Simulation 1 results: error of roll estimates and the estimated trajectory. (**a**) Error of roll estimates of the sine rotation vector method and EKF; (**b**) Estimated trajectory on the xy plane by the sine rotation vector method and EKF.

Table 3 shows the error statistics for the simulation tests. The average and root mean square (RMS) of the attitude estimation error in roll, pitch and yaw are shown together with the distance error ratio relative to the traveled distance. In the table, the elements of a vector are ordered roll, pitch and yaw. In both simulations, the proposed method performs better than the other methods. Because the measurement data of the magnetic field for Simulation 2 are biased less than the data for Simulation 1 (while the measurement data of acceleration for Simulation 2 are biased a little more than the data for Simulation 1), most methods work better in Simulation 2 than in Simulation 1. Figure 2 depicts the roll error and estimated trajectory for Simulation 1. The robot starts at the origin and stops at the destination (83.75,−3.49,4.61) m.

### 6.2. Experiment in a Test Tank

**Figure 3:**
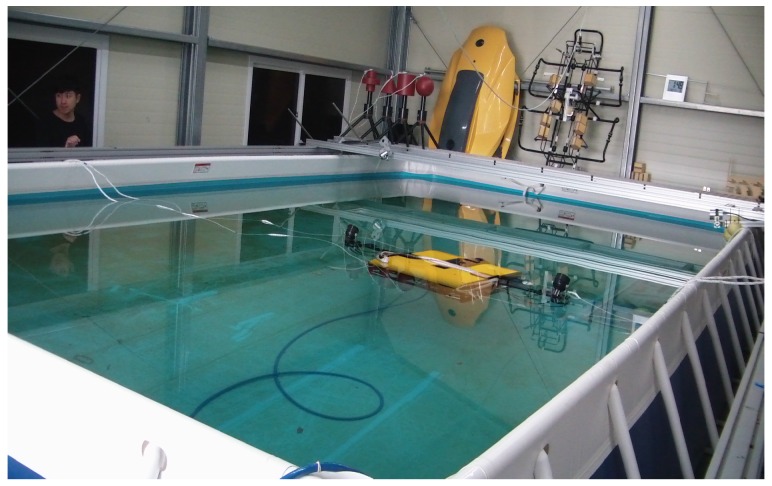
Test tank.

**Figure 4:**
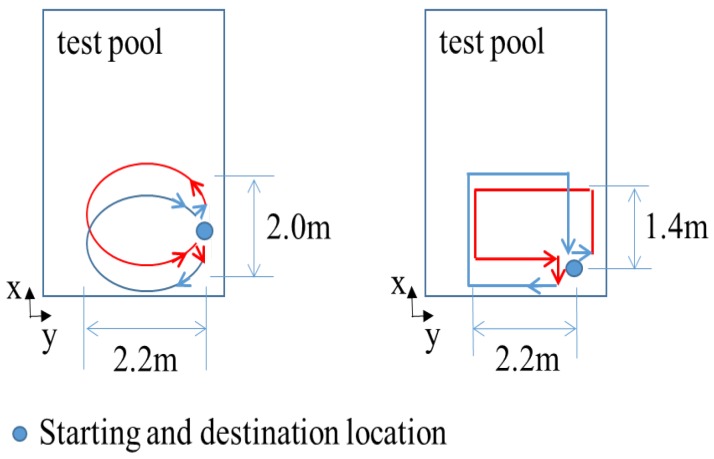
The two trajectories for the navigation experiment.

The environment for the test-tank experiments is shown in Figure 3. The experiments involved the use of an AHRS (Microstrain 3DM-GX3 25) [24] and a DVL (LinkQuest NavQuest 600 Micro) [25]. The robot navigated through the circular and rectangular trajectories shown in Figure 4. For each trajectory, once the robot circulates and returns to its initial location, it turns back and traces the path to the initial location in a reverse direction. One of the problems with the test tank experiment is that the navigation range is limited, and the performance of the method in practical use is not fully verifiable. In order to mitigate the limitation, the test tank experiment adopted turn around and trace back motion. In the experiment, the robot repeats round trip circulation to extend the navigation range, so that the traveled distance is 282.7 m and 100.8 m for the circular and rectangular motion, respectively. In addition, once the robot circulates and returns to its initial location, it turns back and traces the path to the initial location in the reverse direction. This large and sharp turning motion provides a harsher and more serious circumstance to attitude estimation. The robot finally returns to its initial location; thus, the destination of the navigation is the robot’s initial location.

Because the experiment does not involve the use of any instrument that can provide the true attitude, the performance of the methods was verified indirectly from the estimated trajectories that are calculated using the estimated attitude. The estimated location deviates from the true location, and the deviation increases as the travel time and distance are extended. The deviation at the destination reflects the performance of the attitude estimation method; therefore, the distance error between the estimated destination and the actual destination is used as an index for the performance evaluation. Table 4 presents a comparison of the distance errors of the four methods. The distance error ratio indicates the ration of the distance error at the destination relative to the overall traveled distance. In Table 4, “SRV” denotes the method proposed in this paper, and the results show that it outperforms the other methods.

**Figure 5:**
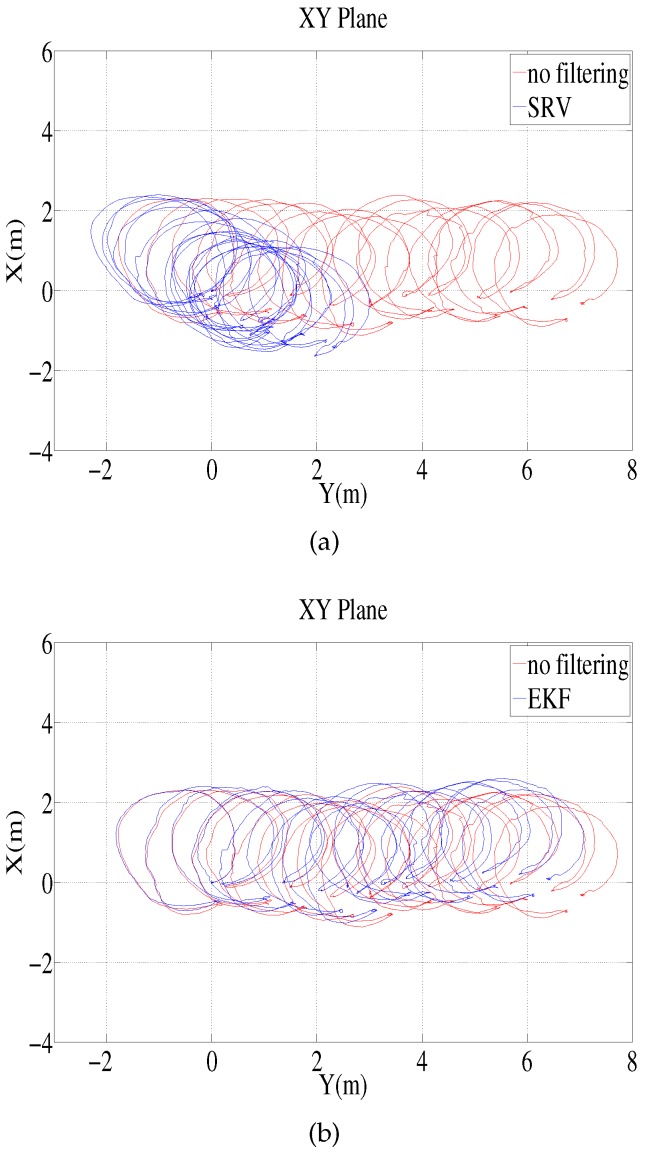
Circular path test tank experiment: trajectory in the xy plane estimated by the proposed method compared to the trajectories calculated without filtering and with the pure-EKF method. (**a**) Trajectory estimated by the proposed method and that calculated without filtering; (**b**) Trajectory estimated by the pure-EKF method and that calculated without filtering.

**Figure 6:**
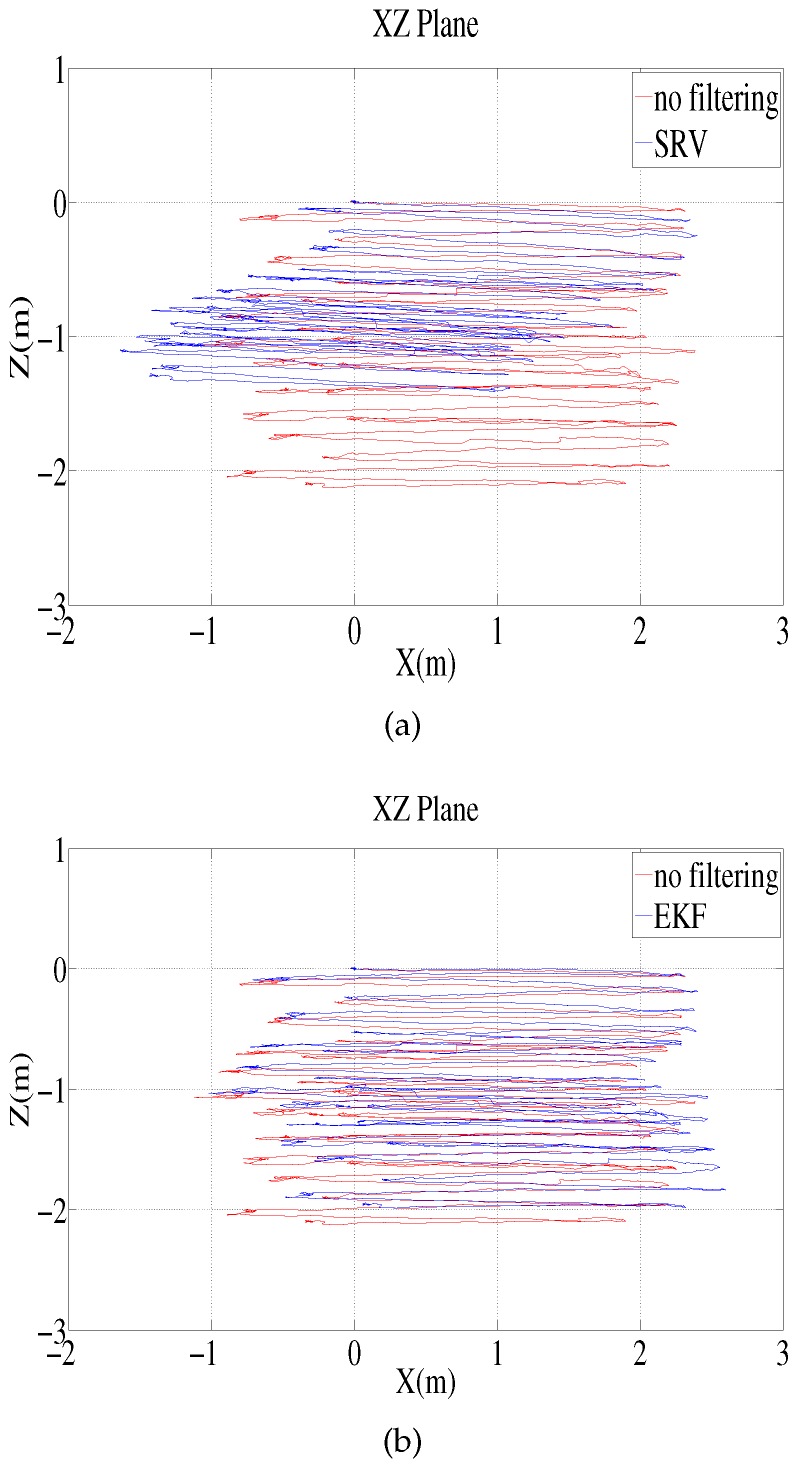
Circular path test tank experiment: trajectory in the xz plane estimated by the proposed method compared to the trajectories calculated without filtering and with the pure-EKF method. (**a**) Trajectory by the proposed method and that calculated without filtering; (**b**) Trajectory by the pure-EKF method and that calculated without filtering.

Figure 5 shows the estimated trajectory for the SRV method. The blue line depicts the estimated trajectory, while the red line indicates the trajectory provided by dead reckoning, whereby the velocity is integrated (without any filtering) to estimate the attitude. Figure 6 shows the results of the pure EKF method, which uses Equations (24), (25) and (27) for the Euler angle measurement. As in Figure 5, the blue line is the estimated trajectory of the EKF, while the red line is the trajectory provided by dead reckoning without filtering.

### 6.3. Experiment on the Ground

**Figure 7:**
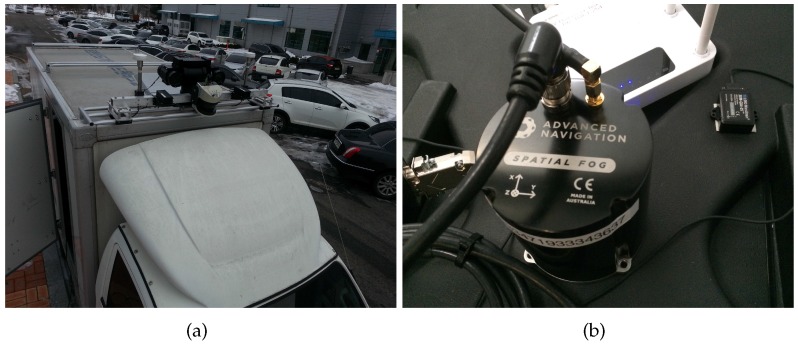
Sensors for the test on the ground. GPS is used to calculate the velocity of the vehicle. (**a**) Sensors on the roof of the test vehicle; (**b**) Fiber optic gyroscope (FOG) and attitude heading and reference system (AHRS) inside the vehicle.

**Figure 8:**
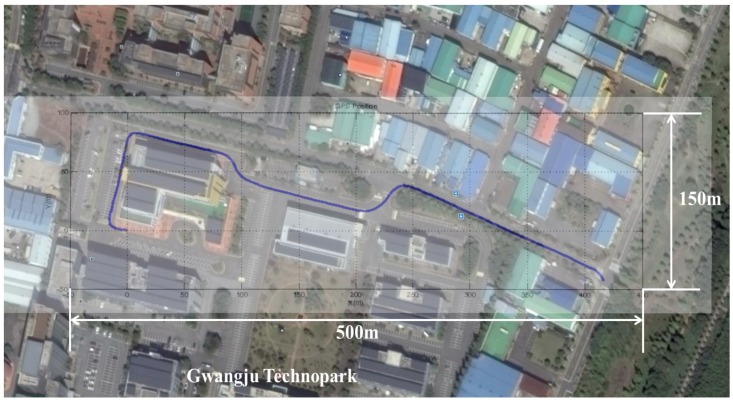
Route for the ground experiment.

The experiment on the ground to test the proposed method uses a ground vehicle equipped with GPS, AHRS (Microstrain 3DM-GX4 25) and FOG. The model’s FOG (Spatial FOG by Advanced Navigation) [26] provides the attitude data used as the reference to compare the attitudes estimated by the methods. Figure 7 shows the vehicle and sensors used for the experiment. The vehicle navigated for 170 s over a distance of approximately 551 m. Figure 8 shows the route of the navigation.

**Figure 9:**
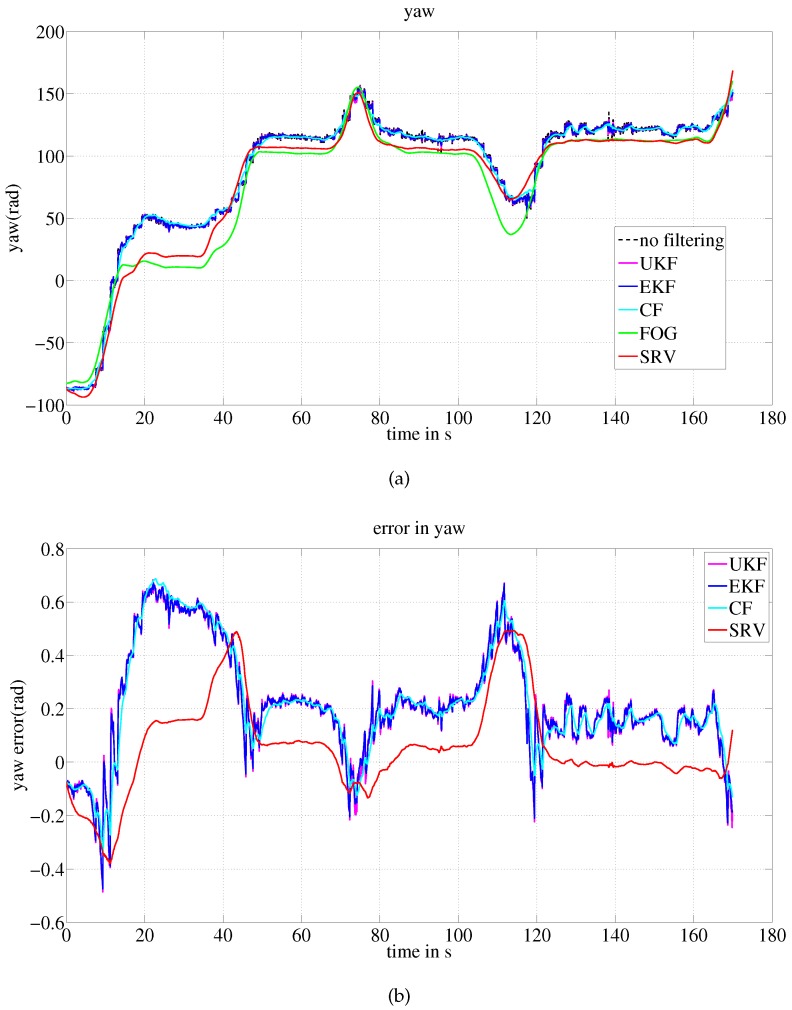
Ground test results: yaw estimates compared to the FOG measurement. (**a**) Yaw estimates of the methods; (**b**) Error of the estimate with respect to the yaw by FOG.

Figure 9 shows the yaw estimates of the methods compared to the yaw provided by FOG, as well as the error of the estimates. The results of the experiment are listed in Table 5, where the statistics of the yaw error are shown. EKF, UKF and CF exhibit comparable error statistics, while the proposed method outperforms these methods.

## 7. Conclusions

This paper proposes a new method for estimating the attitude of an underwater robot. The method uses the sine rotation vector to determine the rotational difference between the measured attitude and the presumed attitude. Compared to existing methods that directly calculate the Euler angle difference, the proposed method resolves the problem that arises from a non-unique mapping between the rotation and the Euler angle difference, when the attitude is described by the parameters of the Euler angles.

The experiments compared the proposed method to the existing methods that use EKFs, UKFs and CFs based on the differences of the Euler angles. The proposed method outperforms all of the other methods in terms of the location estimation of an underwater robot and the attitude estimation of a ground vehicle.

An investigation especially through underwater experiment to compare the properties of the sine rotation vector with other attitude representation methods, such as Euler angles, rotation matrices and quaternions with respect to attitude estimation, remains as the focus of further research. Furthermore, fusing more sensor measurements that provide directional information in the framework of the sine rotation vector is expected. Finally, it is expected that the proposed method will be implemented for navigation in a lake or ocean environment for the verification of the practical applicability, in a future study.

## Figures and Tables

**Table 1:** Complementary aspects of the measurements. DVL, Doppler velocity log.

	Short-Term Use	Long-Term Use
Angular rate (gyroscope)	prediction	-
Gravitational field (accelerometer)	-	correction
Magnetic field (magnetometer)	-	correction
Velocity (DVL)	-	(1) Location estimation
		(2) Rejection of dynamic effect
		in gravitational field measurement

**Table 2:** Uncertainty in the measurement in the simulation tests.

Simulation	Measurement	Uncertainty	Value (degree)
Simulation 1	Acceleration	bias (Δϕ,Δθ,Δψ)	(5,5,1)
		random noise $\left(\sigma_{\phi },\sigma_{\theta },\sigma_{\psi }\right)$	(1,1,1)
	Magnetic field	bias (Δϕ,Δθ,Δψ)	(2,2,5)
		random noise $\left(\sigma_{\phi },\sigma_{\theta },\sigma_{\psi }\right)$	(1,1,1)
Simulation 2	Acceleration	bias (Δϕ,Δθ,Δψ)	(5,5,5)
		random noise $\left(\sigma_{\phi },\sigma_{\theta },\sigma_{\psi }\right)$	(1,1,1)
	Magnetic field	bias (Δϕ,Δθ,Δψ)	(1,1,1)
		random noise $\left(\sigma_{\phi },\sigma_{\theta },\sigma_{\psi }\right)$	(1,1,1)

**Table 3:** Simulation test results: statistics of the estimation error. CF, complementary filter.

Simulation	Statistics	SRV	EKF	UKF	CF
Simulation 1	Average (rad)	$\left(\begin{array}{c} 0.0622\\ 0.0657\\ 0.0570 \end{array}\right)$	$\left(\begin{array}{c} 0.0797\\ 0.0799\\ 0.0571 \end{array}\right)$	$\left(\begin{array}{c} 0.0890\\ 0.0820\\ 0.0600 \end{array}\right)$	$\left(\begin{array}{c} 0.0804\\ 0.0798\\ 0.0557 \end{array}\right)$
	RMS (rad)	$\left(\begin{array}{c} 0.0739\\ 0.0789\\ 0.0599 \end{array}\right)$	$\left(\begin{array}{c} 0.0881\\ 0.0883\\ 0.0606 \end{array}\right)$	$\left(\begin{array}{c} 0.1038\\ 0.0959\\ 0.0693 \end{array}\right)$	$\left(\begin{array}{c} 0.0891\\ 0.0883\\ 0.0600 \end{array}\right)$
	distance error ratio (%)	0.723	1.618	1.689	1.639
Simulation 2	Average (rad)	$\left(\begin{array}{c} 0.0569\\ 0.0614\\ 0.0234 \end{array}\right)$	$\left(\begin{array}{c} 0.0893\\ 0.0876\\ 0.0272 \end{array}\right)$	$\left(\begin{array}{c} 0.1297\\ 0.1206\\ 0.0429 \end{array}\right)$	$\left(\begin{array}{c} 0.1610\\ 0.1236\\ 0.0303 \end{array}\right)$
	RMS (rad)	$\left(\begin{array}{c} 0.0683\\ 0.0744\\ 0.0280 \end{array}\right)$	$\left(\begin{array}{c} 0.1068\\ 0.1050\\ 0.0334 \end{array}\right)$	$\left(\begin{array}{c} 0.1614\\ 0.1512\\ 0.0569 \end{array}\right)$	$\left(\begin{array}{c} 0.2420\\ 0.1767\\ 0.0391 \end{array}\right)$
	distance error ratio (%)	0.245	1.490	1.489	0.617

**Table 4:** Test tank experiment result: the ratio of the distance error relative to the distance traveled.

Trajectory	Distance Error Ratio			
	SRV	EKF	UKF	CF
Circular trajectory	1.08%	2.38%	2.68%	3.37%
Rectangular trajectory	2.06%	4.07%	4.82%	4.82%

**Table 5:** Ground test estimation error: errors in yaw.

Statistics	Error (Rad)			
	SRV	EKF	UKF	CF
Average	0.1264	0.2494	0.2505	0.2476
Root mean square error	0.1862	0.2994	0.3001	0.3016
Maximum error	0.4931	0.6749	0.6826	0.6877
